# Protein Disulfide Isomerase A4 Is Involved in Genome Uncoating during Human Astrovirus Cell Entry

**DOI:** 10.3390/v13010053

**Published:** 2020-12-31

**Authors:** Nayeli Aguilar-Hernández, Lena Meyer, Susana López, Rebecca M. DuBois, Carlos F. Arias

**Affiliations:** 1 Departamento de Genética del Desarrollo y Fisiología Molecular, Instituto de Biotecnología, Universidad Nacional Autónoma de México, Cuernavaca CP 62210, Mexico; nayeliaguilar27@gmail.com (N.A.-H.); susana@ibt.unam.mx (S.L.); 2 Department of Biomolecular Engineering, University of California, Santa Cruz, CA 95064, USA; lmeyer1@ucsc.edu (L.M.); rmdubois@ucsc.edu (R.M.D.)

**Keywords:** astrovirus, protein disulfide isomerase, virus entry

## Abstract

Although human astroviruses (HAstVs) are important agents of gastroenteritis in young children, the studies aimed at characterizing their biology have been limited, in particular regarding their cell entry process. It has been shown that HAstV serotype 8 enters human cells by a classical clathrin-mediated endocytosis pathway; however, the cell receptor or other cell entry factors that may be relevant for an efficient viral infection are unknown. In this work we used a far-Western blotting approach to identify cellular proteins that interact with the recombinant capsid spike proteins of HAstV serotypes 1, 2, and 8, synthesized in *Escherichia coli*. We identified the 72 kDa protein disulfide isomerase A4 (PDIA4) as a binding partner for HAstV-1 and -8 spikes, but not for the HAstV-2 spike. In agreement with this observation, the PDI inhibitor 16F16 strongly blocked infection by HAstV serotypes 1 and 8, but not serotype 2. RNA interference of PDIA4 expression selectively blocked HAstV-8 infectivity. We also showed that the PDI activity does not affect virus binding or internalization but is required for uncoating of the viral genome.

## 1. Introduction

Human astrovirus (HAstV) is an important etiological agent of gastroenteritis, affecting mainly children, the immunocompromised, and the elderly [1]. Most of these infections are associated with the so-called canonical or classic HAstVs, which comprises eight different serotypes (HAstV-1 to -8), with HAstV-1 being the most prevalent worldwide [1]. In recent years, new strains have also been reported as causing meningitis and encephalitis in immunosuppressed patients, increasing interest in the study of these viruses [2].

HAstVs are small, nonenveloped viruses with a single-stranded positive-sense RNA genome of about 6.8 kb. The capsid of the mature, infectious virus has an icosahedral morphology of T = 3 and is composed of two proteins: VP34, which forms the shell of the virus particle (core protein), and VP27, a protein that constitutes the 30 dimeric globular spikes that protrude from the virion (spike protein) [3]. The spike protein, but not the core protein, has been shown to induce neutralizing antibodies [4] and several neutralizing antigenic determinants have been mapped on the spike, defined either by X-ray crystallography [5] or by sequencing HAstV-1, -2, and -8 mutants that escape neutralization by monoclonal neutralizing antibodies [4]. In addition, the HAstV spike has been shown to specifically bind to the surface of Caco-2 cells and to contain the receptor-binding domain [5].

HAstV has been shown to enter cells by clathrin-mediated endocytosis [6]; however, very little is known about the viral receptor or other cell factors that may determine virus entry. In this work we took advantage to the fact that the astrovirus capsid core and spike proteins can be successfully produced in *Escherichia coli* while maintaining their native structure [4,7,8] to identify interacting cellular proteins relevant for HAstV infection using a far-Western blotting approach. We found protein disulfide isomerase A4 (PDIA4) is an entry factor associated with genome uncoating of HAstV serotypes 1 and 8.

## 2. Material and Methods

### 2.1. Cells and Viruses

Human colorectal adenocarcinoma cells Caco-2, clone Bbe1 (ATCC, Manassas, VA, USA; CRL-2102), were cultured in high-glucose Dulbecco’s modified Eagle’s medium (DMEM-HG) supplemented with 10% fetal bovine serum (FBS). African green monkey kidney epithelial MA104 (ATCC, Manassas, VA, USA; CRL-2378), Vero (ATCC, Manassas, VA, USA; CCL-81), and Madin-Darby canine kidney (MDCK II) cells (ATCC, Manassas, VA, USA; CRL-2936) were grown in DMEM containing 5% FBS. Human astrovirus serotypes 1 (HAstV-1, strain Oxford), 2 (HAstV-2, strain Oxford) and 8 (HAstV-8, strain Yuc-8) were grown in Caco-2 cells [4]. Stocks of HAstV-1, HAstV-2 and HAstV-8 were prepared in Caco-2 cells, as previously described [9]. Briefly, the virus was activated with 200 µg/mL of trypsin for 1 h at 37 °C, and soybean trypsin inhibitor (Sigma, St. Louis, MO, USA) at 200 µg/mL was added immediately before inoculation of cells. The virus was adsorbed for 1 h at 37 °C, and after this time the inoculum was removed and the infection was left to proceed for 16 h at 37 °C.

### 2.2. Virus Infectivity Assay

The infectious titer of the virus stocks was determined in monolayers of Caco-2 cells grown in 96-well plates. For this, cells were infected as described above with serial two-fold dilutions of the virus stock, and after 16 h of incubation at 37 °C, the cells were fixed with 2% paraformaldehyde in PBS for 20 min at room temperature (RT) and permeabilized by incubation with 2% Triton X-100 in PBS for 15 min at RT. The viral antigen was detected with serotype-specific polyclonal antibodies raised to the capsid spike protein of HAstV-1 (rabbit anti-spike1; dil. 1:1000), -2 (mouse anti-spike2; dil. 1:200) or -8 (rabbit anti-HAstV Yuc8; dil. 1:2000), as described [4], followed by incubation with the corresponding species-specific peroxidase-conjugated antibodies (KPL, Gaithersburg, MD, USA) diluted 1:3000. The focus forming units (ffus) were visually counted in in a Nikon TMS inverted phase-contrast microscope with a 20× objective.

### 2.3. Expression and Purification of Recombinant HAstV Capsid Spike Proteins

Expression and purification of recombinant HAstV capsid spike proteins were performed as described previously [8,9]. Briefly, an expression plasmid encoding a HAstV spike in-frame with a C-terminal 10-histidine tag was transformed into *E. coli* strain BL21(DE3), and protein production was induced with isopropyl-β-D-thiogalactopyranoside. *E. coli* cells were lysed by ultrasonication and soluble HAstV spike was purified by metal affinity chromatography. HAstV spike was dialyzed into PBS and further purified by size-exclusion chromatography on a Superdex 200 column in PBS. Synthetic genes codon optimized for *E. coli*-encoded HAstV-2-Oxford capsid spike amino acids 431 to 674 (GenBank accession number KY964327), HAstV-1 capsid spike amino acid 429 to 645 (accession number AAC34717.1), or HAstV-8 capsid spike amino acids 429 to 647 (UniProtKB entry Q9IFX1) were used.

### 2.4. Far-Western Blot Analysis

Cell membrane protein extracts from the different cell lines were prepared using the Qproteome Cell Compartment Kit (Qiagen, Hilden, Germany) according to the manufacturer’s instructions. The proteins in these extracts (around 100 µg per lane) were separated by 10% SDS-polyacrylamide gel electrophoresis (SDS-PAGE) and transferred to nitrocellulose membranes. The nitrocellulose membranes were blocked with 5% skim milk (Carnation, Solon, OH, USA) in PBS-0.1% Tween 20 for 1 h at room temperature, and after this time the membranes were incubated overnight at 4 °C with 100 µg/mL of the corresponding recombinant spike of HAstV serotypes 1, 2 or 8 in 1% skim milk prepared with PBS-0.1% Tween 20, and subsequently washed three times with PBS-0.1% Tween 20. Anti-spike mouse or rabbit polyclonal antibodies generated in our laboratory were added at a 1:1000 dilution and incubated for 1 h at RT. After washing the membranes three times with PBS-0.1% Tween 20, peroxidase-conjugated rabbit or mouse antibodies (KPL, Gaithersburg, MD, USA, dil. 1:3000) were added and incubated for 1 h at RT. Finally, the membranes were washed once and the presence of the astrovirus spike was detected by chemiluminescence using the Western Lightning system (Perkin Elmer, Waltham, MA, USA).

### 2.5. LC-MS

Protein bands in the 70 to 75 kDa range identified in the far-Western assay were cut out from a parallel SDS gel stained with Coomassie brilliant blue R-250 (Sigma, St. Louis, MO, USA), and the polyacrylamide slices were sent to the Proteomic facility at the Instituto de Biotecnología, UNAM (Cuernavaca, Mexico) for their identification by nano-liquid chromatography (nano-LC)-tandem mass spectrometry (MS/MS).

### 2.6. Western Blot

Cellular lysates of siRNA-transfected cells or 300 ng of a recombinant PDIA4 protein (Novoprotein, Fremont, CA, USA; CA58) were separated by 10% SDS-PAGE; the proteins were then transferred to nitrocellulose membranes (Millipore, Bedford, MA, USA) as reported [10]. The membranes were blocked with 5% nonfat milk for 1 h at RT, followed by incubation with anti-PDIA4 monoclonal antibody (ThermoFisher, Rockford, IL, USA; PA1-007; dil. 1:500) for 1 h at RT. The unbound antibody was washed three times with PBS-0.1% Tween 20, and secondary anti-mouse peroxidase-conjugated antibodies, diluted 1:3000 in PBS-0.1% Tween 20, was added and incubated for 1 h at RT. After this time, the membranes were washed three times with PBS-0.1% Tween 20, and the peroxidase activity was detected using the Western Lightning Chemiluminescence Reagent Plus (PerkinElmer Life Sciences, Boston, MA, USA) according to the manufacturer’s instructions.

### 2.7. RNA Interference

Caco-2 cells were transfected with 5 μM of the siRNA pool of either PDIA4 (Dharmacon M-019249-01-0005) or an irrelevant scrambled control (Dharmacon, Lafayette, CO, USA; D-001206-13-05) using oligofectamine reagent (Invitrogen, Carlsbad, CA, USA), according to the manufacturer´s instructions. Seventy-two hours later, the transfection mixture was removed and the cells were washed twice with minimum essential medium (MEM) and infected with HAstV-2 or -8 at a multiplicity of infection (MOI) of 3, as described above. At 18 hpi the cells were harvested in Laemmli sample buffer and the presence of PDIA4 was assessed by 10% SDS-PAGE and Western blotting.

### 2.8. Pharmacological Inhibitors

PDI inhibitors 16F16 (Sigma, St. Louis, MO, USA; SML0021), PACMA 31 (Sigma, St. Louis, MO, USA; SML0838), and di-tert-butyl nitroxide (DTBN, Sigma, St. Louis, MO, USA; 300721) were dissolved in DMSO and used at the indicated concentrations. Caco-2 cells grown in 96-well plates were washed once with MEM and then pretreated with the inhibitors, or DMSO as control, for 1 h at RT. After removal of the drugs, the cell monolayers were washed twice with MEM and then infected with the different serotypes of HAstV at an MOI of 0.025 for 1 h at 37 °C. After 16 hpi the cells were fixed and the virus infectivity was determined by a focus-forming unit (ffu) assay, as described above.

### 2.9. Virus Binding Assay

HAstV-8 virus particles, purified as previously described [11] were added at an MOI of 30 to Caco-2 cell monolayers in 48-well plates that were pretreated or not with the drugs or with siRNAs as described above, for 1 h on ice. The unbound virus was washed three times with cold PBS. Total RNA was extracted with TRIzol reagent (Invitrogen, Carlsbad, CA, USA) according to the manufacturer´s instructions. cDNA was generated by reverse transcription using M-MLV Reverse Transcriptase (Invitrogen, Carlsbad, CA, USA) and qPCR was performed with Real Q Plus Master Mix Green (Ampliqon, Copenhagen, Denmark) using an ABI Prism 7500 detection system (Applied Biosystems, Foster City, CA, USA). The primers used amplify a region of the ORF 1b of HAstV-8; the amplification conditions were previously reported [12]. The amount of viral RNA was normalized to that of 18S RNA [13].

### 2.10. Viral RNA Transfection

Caco-2 cells were infected with HAstV-8 at an MOI of 2 for 16 h at 37 °C. After this time, total RNA was extracted with TRIzol reagent (Invitrogen, Carlsbad, CA, USA) according to the manufacturer´s instructions. The extracted RNA was used to transfect Caco-2 cells previously transfected with PDIA4 siRNAs or pretreated with the PDI inhibitor 16F16. Briefly, Caco-2 cell monolayers at 60–80% confluence in 48-well plates were incubated for 6 h at 37 °C with a mixture of Lipofectamine 2000 (Invitrogen, Carlsbad, CA, USA) and 200 ng of total RNA extracted from HAstV-8-infected Caco-2 cells. After this time, the transfection mixture was removed, the cells were washed twice with MEM, fresh MEM was added, and the cells were incubated for 24 h before staining for the presence of HAstV-8 focus-forming units (ffu’s) using an immunoperoxidase assay, as described above.

### 2.11. Virus Internalization Assay

Confluent Caco-2 cell monolayers in 48-well plates were pretreated with 500 µM of 16F16 for 1 h at RT, and then HAstV-8 at an MOI of 50 was added and the cells were further incubated for 1 h at 4 °C. After this time, the unbound virus was removed by washing the cells three times with PBS and the cell monolayers were switched to 37 °C for 1 h to allow the virus to be internalized. To remove the virus adsorbed to the cell surface that had not been internalized during the incubation period at 37 °C, the cells were incubated with neutralizing monoclonal antibody 2D9 against HAstV-8, diluted 1:20, for 1 h at 4 °C. At this concentration, this antibody is able to remove up to 80% of the cell surface bound virus (unpublished data). Then, the internalized viral RNA was quantified by qRT-PCR as described above.

### 2.12. Viral Uncoating Assay

To determine the moment at which the virus genome is uncoated and released from the endocytic vesicle, a neutral red assay was performed, as previously described [6]. Briefly, neutral red-labeled HAstV-8 virus stock was prepared by growing the virus in Caco-2 cells in the presence of 10 µg/mL neutral red for 18 h at 37 °C protected from light. After this time, the virus was harvested and titrated by an immunoperoxidase assay as described above, under light or in a dark room to confirm its photosensitivity. To evaluate the effect of 16F16 on virus uncoating, confluent Caco-2 cell monolayers were pretreated with the drug for 1 h at RT, then the cells were infected with neutral red-labeled HAstV-8 at an MOI of 0.025 and incubated in the dark for 2 h at 37 °C to allow the virus to enter the cells. After this time the cells were exposed to direct white light for 10 min. After light exposure, the infection was continued without protection from light for 16 h at 37 °C; the cells were then fixed and the infectivity determined by an immunoperoxidase assay.

### 2.13. Statistical Analysis

The statistical significance of the data was evaluated by the Mann–Whitney test using GraphPad Prism 5 (GraphPad Software Inc., San Diego, CA, USA).

## 3. Results

### 3.1. The HAstV-1 and -8 Capsid Spikes Interact with Cellular Proteins of around 70–75 kDa

**Figure 1:**
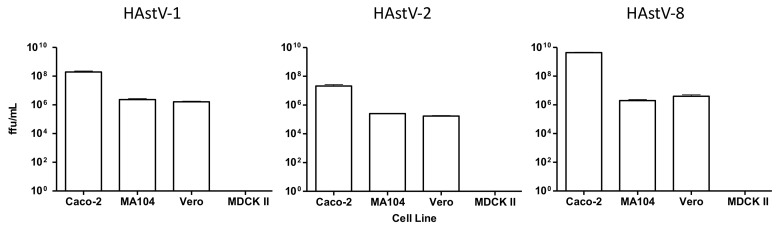
Permissiveness of cell lines to HAstV infection. Caco-2, MA104, Vero and MDCKII cells were infected with serial two-fold dilutions of HAstV-1, HAstV-2 or HAstV-8 and the infectious titer was determined by an immunoperoxidase assay (see Material and Methods). The arithmetic means and SEM for three independent experiments performed in duplicate are shown.

To search for cellular proteins that could bind the HAstV capsid spike and could be potentially involved in virus cell entry we evaluated a far-Western blot assay approach. We tested the spike proteins of three different HAstV serotypes, since some variability in the capacity of different serotypes to productively infect the same cell line has been found [14], suggesting the potential usage of different cell receptors. In addition, we also evaluated four different cell lines that are differently permissive to HAstV infection: Human adenocarcinoma Caco-2 cells (highly permissive), African green monkey kidney MA104 and Vero cells (partially permissive), and Madin-Darby canine kidney MDCKII cells (non-permissive) (Figure 1 and ref. [14]).

**Figure 2:**
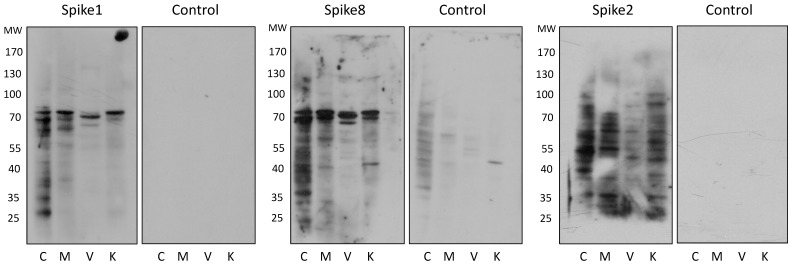
Potential cellular interactors for HAstV spike proteins identified by far-Western blotting. Cell membrane-enriched protein extracts from the indicated cell lines were separated by SDS-PAGE and transferred to nitrocellulose membranes that were incubated overnight with the recombinant spike proteins corresponding to HAstV-1, -2 or -8, and the spike proteins were detected with serotype-specific anti-spike antibodies. As a control, membranes were incubated without the spike and were developed with the antibodies against the respective spike. A representative image from at least three independent experiments is shown. C, Caco-2; M, MA104; V, Vero; K, MDCKII cells.

**Figure 3:**
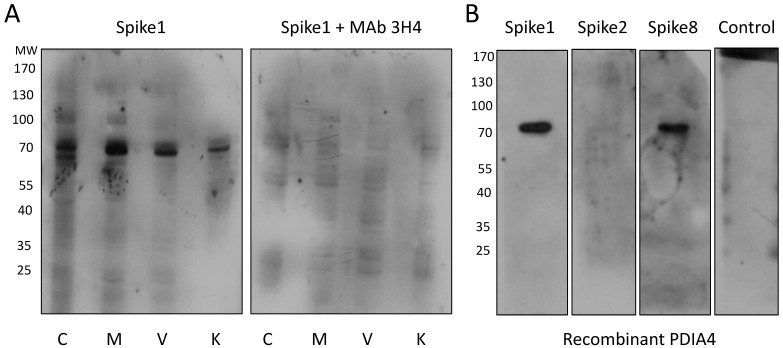
(**A**) A neutralizing monoclonal antibody to HAstV-1 blocks the interaction of spike 1 with the cellular 70–75 kDa proteins. A far-Western assay was performed as described in Material and Methods. Spike 1 was pre-incubated for 1 h at room temperature with or without neutralizing MAb 3H4, then added to the membrane and incubated overnight. The spike bound to cellular proteins was detected with an anti-spike 1 antibody. (**B**) HAstV interacts with PDIA4 in a serotype-specific manner. Recombinant PDIA4 protein (300 ng/lane) was separated by SDS-PAGE and transferred to nitrocellulose membrane and a far-Western assay was performed using the indicated HAstV spikes or without a spike protein as a control. Detection of the corresponding spikes was performed with specific anti-spike antibodies. The molecular weight (MW) is indicated. A representative image from at least three independent experiments is shown. C, Caco-2; M, MA104; V, Vero; K, MDCKII cells.

For the far-Western analysis, cell membrane-enriched protein extracts were prepared, separated by SDS-PAGE, and transferred to nitrocellulose membranes. The membranes were then incubated with the recombinant capsid spike protein of HAstV serotype 1, 2 or 8 (spike 1, 2 or 8). The corresponding spike protein was detected with polyclonal antibodies specifically raised to each of the three recombinant proteins [4,9]. Spikes 1 and 8 were found to interact consistently, and most prominently, with two cellular proteins that migrated in the range of 70 to 75 kDa, while in the control lanes, incubated only with the anti-spike antibody in the absence of the spike proteins, these bands were not observed (Figure 2). Although slight variations in the electrophoretic migration of these two bands were observed in the different cell lines, they were identified in all cells tested (permissive, semi-permissive and non-permissive). Of interest, spike 2 does not seem to interact with the 70–75 kDa proteins recognized by the spike proteins of serotypes 1 and 8, suggesting that HAstV-2 might recognize different molecules on the cell surface. In these assays, several bands of lower molecular weight, not present in the control conditions, were observed when the cell membrane extracts were incubated with either of the three spikes. The presence of these bands was not reproducible in different experiments, although a potential role of some of these proteins in the interaction of the viruses with the cell surface cannot be discarded. Interestingly, when the spike 1 protein was pre-incubated with the neutralizing MAb 3H4, which inhibits the binding of HAstV-1 to the cell surface (unpublished data), its binding to the 70–75 kDa bands was clearly abolished (Figure 3A).

### 3.2. The HAstV- 1 and -8 Capsid Spikes Interact with PDIA4

Given the consistent detection of the 70–75 kDa protein bands by spike 1 and 8 proteins, the identity of the bands was explored by excision of this region of the gel and analysis of the proteins by liquid chromatography coupled with tandem mass spectrometry (LC-MS/MS). A total of 30 typical plasma membrane proteins or proteins that have been described to be present at the plasma membrane were identified in Caco-2 cells, 11 of which have been described as virus receptors (Table S1). Protein disulfide isomerase family A member 4 (PDIA4) was found in all four cell lines analyzed. In addition, since the molecular weight of PDIA4 (72 kDa) fits well in the range of the protein bands detected with spikes 1 and 8, we selected it for further characterization. To establish if there was an interaction between spikes 1 or 8 and PDIA4, we analyzed whether these proteins bound in a far-Western blot analysis in which a recombinant PDIA4 protein was loaded in a SDS-PAGE and blotted. We observed a specific interaction of spikes 1 and 8 with PDIA4, whereas neither spike 2 nor the core protein of HAstV-1, which were used as negative controls, recognized the PDIA4 protein (Figure 3B). These findings strongly suggest that PDIA4 might be one of the binding partners for spikes 1 and 8 originally identified in the far-Western assay and also indicates that the interaction of the capsid spike of HAstV with PDIA4 is serotype-specific.

### 3.3. PDI Activity is Important for HAstV-1 and HAstV-8 Infection

**Figure 4:**
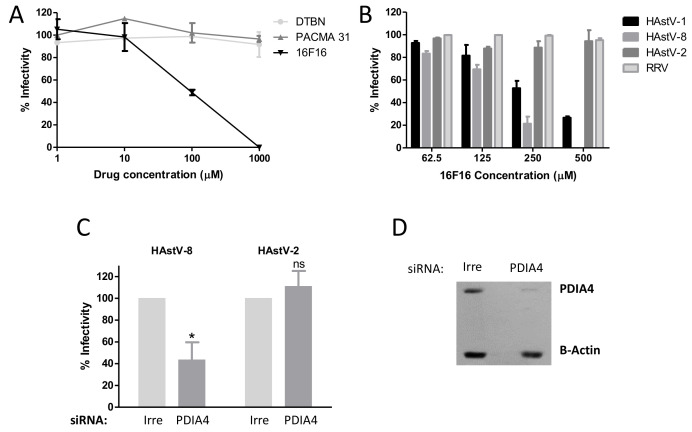
PDIA4 is important for astrovirus infection. (**A**) Caco-2 cells were incubated with the indicated PDI inhibitors for 1 h at 37 °C, the cells were washed and infected with HAstV-8 and 16 h post-infection (hpi), and the viral infectivity was determined by an immunoperoxidase assay as described in Material and Methods. (**B**) Caco-2 cells were pre-treated for 1 h at 37 °C with the indicated concentrations of PDI inhibitor 16F16. Cells were washed and infected with either HAstV-1, -2, -8 or with rhesus rotavirus (RRV), and the viral infectivity was determined. Data in A and B are expressed as percentage of viral infectivity compared to control cells treated with DMSO, which was taken as 100%. (**C**) Caco-2 cells were transfected with an siRNA pool directed to PDIA4 or with an irrelevant (Irre) siRNA pool, and 72 h post-transfection (hpt) cells were infected with the indicated HAstV strain (MOI = 0.025). At 16 hpi, the cells were fixed, and the viral infectivity was determined as described previously. Virus infectivity is expressed as the percentage of infected cells obtained in the control-transfected cells (Irre), which was taken as 100%. The arithmetic mean ± SEM from three independent experiments performed in duplicate is shown. * *p* < 0.05. (**D**) A representative Western blot showing the effect of the siRNA-PDIA4 on the expression of PDIA4 as compared to cells transfected with an irrelevant siRNA. β-Actin was used as a loading control.

To explore the role of PDIA4 in viral infection, Caco-2 cells were pre-treated for 1 h at 37 °C with different pharmacological PDI inhibitors that target the cysteines at the active site of the enzyme. Following infection with HAstV-8, the effect of the PDI inhibitors on viral infectivity was determined by an immunoperoxidase assay as previously described [15]. Of the 3 inhibitors tested (DTNB, 16F16 and PACMA31) only 16F16, a cell permeable inhibitor that had been reported to inhibit the enzymatic activity of PDIA4 [16], showed an inhibitory effect on HAstV-8 infection (Figure 4A). HAstV-1 was also affected by this drug, with a 75% reduction of infectivity at 500 µM, while the infectivity of HAstV-2 was not affected by this drug (Figure 4B). As a control we also tested the effect of 16F16 on a rhesus rotavirus (RRV) strain; the infectivity of this virus was not decreased in the presence of the inhibitor (Figure 4B), indicating that the effect is virus-specific as well as HAstV serotype-specific. The viability of the cells, as determined by an LDH assay, was not affected, even at the highest concentrations tested (data not shown). Furthermore, knocking-down the expression of PDIA4 in Caco-2 cells by transfection with a pool of specific siRNAs directed to its mRNA (Figure 4D) reduced the infectivity of HAstV-8 by about 60% as compared to a control transfection with an irrelevant scrambled siRNA, indicating the involvement of PDIA4 in the infection of this virus (Figure 4C); on the other hand, as expected, the infectivity of HAstV-2 was not affected by the siRNAs. Altogether, these findings suggest that the activity of PDIA4 is relevant for HAstV-1 and -8 infection, but not for HAstV-2, consistent with the results obtained in the far-Western assays.

### 3.4. PDI Has a Role during HAstV-8 Cell Entry

**Figure 5:**
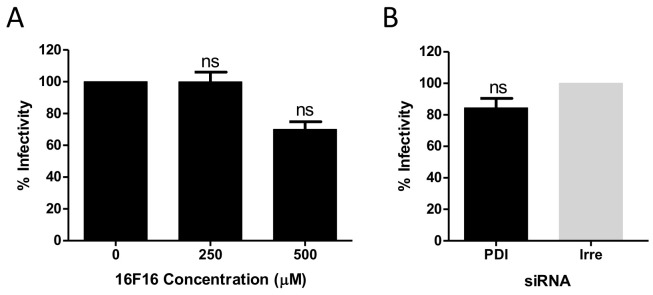
PDIA4 is not required for astrovirus replication when the entry step is bypassed by transfection of the viral genomic RNA. Caco-2 cells were transfected with 200 ng of total RNA extracted from HAstV-8 infected cells that were previously (**A**) treated or not with the indicated concentrations of 16F16 or (**B**) transfected either with the PDIA4 siRNA or with an irrelevant siRNA for 72 h. After 24 h post-transfection, the infectivity was determined as described in Material and Methods. Virus infectivity is expressed as the percentage of infected cells obtained in the control cells treated with DMSO or that were transfected with the irrelevant siRNA, which were taken as 100%, respectively. The arithmetic means ± SEM from three independent experiments performed in duplicate is shown; ns, non-significant.

To determine the step at which PDI has a role during HAstV infection, we took advantage of the fact that the genome of single-stranded, positive-sense RNA viruses can be transfected into permissive cells, leading to a productive infection that skips the entry step. Thus, total RNA was purified from HAstV-8-infected Caco-2 cells and was used to transfect cells that had been pre-treated with 16F16. We did not find an inhibitory effect at 250 µM, and observed a non-significant, slight inhibition of about 30% of infectivity in the presence of 500 µM of the drug (Figure 5A), a concentration that strongly reduced the infectivity of HAstV-8 (Figure 4B). In addition, when the viral RNA was transfected into cells where the expression of PDIA4 had been knocked-down, no reduction in the infectivity titer was observed, as compared to cells previously treated with an irrelevant siRNA (Figure 5B). These results indicate that the presence and activity of PDIA4 is not likely relevant for virus infection steps following genome uncoating.

### 3.5. PDI Is Required for Uncoating of HAstV-8 during Cell Entry

**Figure 6:**
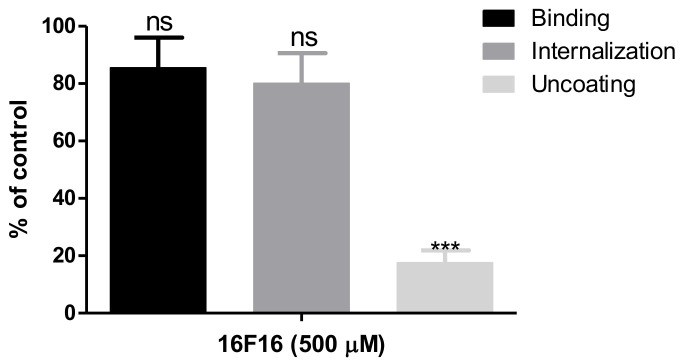
HAstV-8 requires PDI for uncoating during virus infection. Caco-2 cells were pre-treated with 16F16 (500 µM) for 1 h at room temperature. Then, viral binding, internalization, and uncoating assays were performed as described under Material and Methods. Data are expressed as percentage of the cell-bound (first bar) or internalized (second bar) virus, as determined by RT-qPCR, as compared to cells treated with DMSO, which was taken as 100%. The third bar represents the percentage of viral infectivity observed of the neutral red-labeled virus exposed to light as compared to the same virus kept in the dark. The arithmetic means ± SEM from three independent experiments performed in duplicate is shown. *** *p* < 0.001.

To further delineate the particular step during which PDIA4 is required for the virus cell entry process, a virus binding assay was carried out in Caco-2 cells pretreated with 16F16. In these assays, purified HAstV-8 at an MOI of 10 was added to cells that were pretreated for 1 h with the PDI inhibitor, and was incubated for 1 h on ice to prevent virus entry. The unbound virus was then removed, total RNA was extracted, and the viral RNA was quantified by RT-qPCR. We found that PDIA4 activity was not required for this initial step of the virus–cell interaction, since attachment of the virus to the cell surface was not prevented in cells pre-treated with up to 500 µM of inhibitor (Figure 6).

Since attachment of the virus to the cell surface was not affected under conditions that greatly reduce HAstV-8 infectivity, we evaluated if internalization of the virus particles was perturbed in the presence of the drug. For this, Caco-2 cells were treated with 500 µM 16F16 for 1 h at room temperature, and HAstV-8 at an MOI of 50 was adsorbed to the cell surface for 1 h at 4 °C. After this time, the cells were shifted to 37 °C for 1 h to allow the bound virus to be endocytosed, and the non-internalized virus was removed from the cell surface by further incubation of the cells for 1 h at 4 °C with MAb 2D9 directed to HAstV-8, which has been shown to detach 80% of the surface-adsorbed virus under these conditions (unpublished data). The internalized viruses were then quantified by qRT-PCR. No effect on virus internalization was detected by treatment of cells with 16F16 when compared to untreated, control cells (Figure 6).

Considering the previous results, we tested whether the PDI activity could be required for the final step of the virus entry process, i.e., uncoating of the viral genome that occurs when the virus in the endocytic vesicle liberates the viral genetic material into the cytoplasm. For this, a neutral red-based RNA release assay was carried out, as described [6]. This assay is based on the capacity of the dye to be incorporated into the viral particles during replication of the virus. The proximity of neutral red to the viral RNA makes the virus infectivity sensitive to exposure to light; however, once the genomic RNA is released into the cytoplasm, the dye diffuses out and the infection can take place in the light [17]. Based on this premise, we prepared a virus stock by infecting Caco-2 cells in the presence of neutral red, in the dark, which was then used to infect 16F16-treated or untreated Caco-2 cells in the dark for 2 h at 37 °C to allow the virus to be internalized. After this time the cells were removed from the dark and the infection was left to proceed for 16 h at 37 °C under regular white light conditions. The infectivity was determined 16 h post-infection by an immunoperoxidase assay. If virus uncoating is affected in the presence of 16F16, the viral infectivity would be reduced when the drug-treated cells are exposed to light after the internalization process has occurred. Of interest, the infectivity of HAstV-8 was decreased by about 80% under these conditions at 500 µM 16F16 (Figure 6), strongly suggesting that the activity of PDI facilitates the exit of the virus from the endocytic vesicle and the release of the viral genome into the host cytoplasm.

## 4. Discussion

During the process of cell entry, viruses must recognize cellular receptors present on the surface of the host cell but also frequently interact with additional cell molecules that facilitate the internalization of the viral particles and/or the delivery of transcription-competent virus structures, which range from the naked virus genome to subviral particles.

We have previously reported that HAstV-8 enters Caco-2 cells using a clathrin-dependent pathway, and drugs that reduce the cholesterol in the cell membrane or that inhibit actin polymerization decrease astrovirus infectivity [6]; however, the cellular receptor for these viruses has not yet been identified, although initial binding of the virus to glycans on the cell surface has been suggested [6,7]. A virus overlay protein binding assay has been used to characterize putative cellular receptors of different viruses, including adenovirus [18], Japanese encephalitis virus [19], nervous necrosis virus [20], Newcastle disease virus [21], and rotavirus [22]. In this work, considering the availability of a recombinant capsid spike protein that mimics the virus domain through which HAstV attaches to the cell surface [5], we used a far-Western approach to identify cell membrane proteins that could potentially interact with the virus during cell entry. Using this approach we found two distinctive cellular proteins, in the range of 70 to 75 kDa, that were recognized by spikes of HAstV serotypes 1 and 8. Of interest, these two bands, with slight variations in their molecular weight, were recognized in permissive as well as in nonpermissive MDCKII cells, indicating that if this protein is the homolog in the various cell lines tested, this interaction might not be the one that confers specificity of the virus to infect Caco-2 cells.

Analysis by LC-MS/MS of proteins in the 70–75 kDa range recovered from preparative SDS-polyacrylamide gels identified several membrane proteins of interest which have been previously reported as cell entry factors for different viruses (Table S1). Out of these, we chose to further characterize PDIA4 (also known as Erp72), a 72 kDa member of a protein family that catalyzes the formation, breakage, and rearrangement of disulfide bonds within protein molecules. Initially, a pharmacological approach was employed, testing several inhibitors of PDI activity. We found that 16F16 strongly decreased HAstV-1 and -8 infectivity in a dose-dependent manner, while it did not affect the infectivity of HAstV-2. These findings are consistent with the different binding patterns observed in the far-Western assay for spike 2 as compared to spikes 1 and 8, indicating a serotype-specific effect of the drug on HAstV infection. The specificity of the drug action is supported by the fact that, similarly to HAstV-2, the inhibitor did not affect the infectivity of rotavirus RRV, which belongs to a different virus family. It is interesting that the other PDI inhibitors tested did not inhibit HAstV-1 or -8 infection; probably because the tested drugs have different biochemical properties and mechanisms of action. Importantly, 16F16 has been shown to be permeable to cells, contrasting with DTBN, which is impermeable [23], suggesting that the internalization of the drug into the cells is important. This is true for influenza infection, where the permeability of PDI inhibitors correlated with their antiviral activity [24]. Furthermore, the selectivity of each drug for its PDI target has to be considered; while PACMA 31 has been reported to be more selective for PDIA1 (57 kDa) [25], 16F16 is able to interact with other PDI family proteins as PDIA6 (48 kDa) and PDIA4 (72 kDa; Erp72) [16]. The effect of silencing the expression of PDIA4 using a pool of siRNAs directed to its mRNA further confirmed the role of this protein in the HAstV infection cycle, although it cannot be discarded that 16F16 may target PDI enzymes in addition to PDIA4 that may play a role in HAstV infection.

Cell surface PDI enzymatic activity has been associated with the cell entry of dengue virus [26,27], Sindbis virus [28], Newcastle disease virus [29], and human immunodeficiency virus [30]. PDI may directly interact and modify viral proteins during this process, as reported for human immunodeficiency virus and the Newcastle disease virus, since their fusion proteins undergo a disulfide bond reduction by PDI activity, inducing conformational changes that allow the virus to enter the cell [29,30,31]. The activity of PDI may also indirectly participate in virus entry; in the case of dengue virus it has been reported that PDI inhibitors prevent virus entry into endothelial cells by blocking the activation of integrins, a requirement for virus infection [28], although the possibility of a direct PDI effect on dengue virus proteins cannot be excluded. Of interest, PDI inhibitors decrease Newcastle disease virus and human immunodeficiency virus cell entry, although binding of these viruses to their receptor is not affected [29,30]. Similar to these observations, in this study we found that the 16F16 PDI inhibitor does not prevent the attachment of the virus to the cell surface or its internalization, but rather it seems to affect virus uncoating. These findings indicate the PDI enzymatic activity in HAstV-1 and -8 infection is required at a post-attachment step.

Whether the HAstV capsid proteins undergo a conformational change due to the enzymatic or chaperone activity of PDI4 that facilitates virus uncoating or whether the PDI activity modifies the oxidation state of a cellular protein that in turn facilitates HAstV uncoating remains to be determined. Future studies should address whether the spike of some serotypes of HAstV is a substrate for PDI activity and how this activity allows the virus genome to be uncoated and be released into the cell’s cytosol.

## 5. Conclusions

In this work we report the requirement for a protein disulfide isomerase activity, possibly associated with PDIA4, during the cell entry process of HAstV. This requirement was found to be serotype-specific, since a PDI inhibitor blocked the infection of HAstV serotypes 1 and 8 but not that of a serotype 2 virus, and it was determined that PDI has a role during uncoating of the viral genome. It will be of interest to learn about the determinants of sensitivity of some HAstV serotypes to the PDI inhibitor and determine if this trait is common for other classical HAstV serotypes as well as for the two groups of novel HAstVs (MLB and VA/HMO) reported. The PDI enzymatic activity has been described to be important in promoting the structural rearrangement of the fusion protein of several enveloped viruses. It is of interest to determine the mechanism through which PDI favors HAstV genome uncoating, in particular whether the PDI activity directly affects the nonenveloped viral capsid, or if its effect is indirect.

## Data Availability

No new data were created or analyzed in this study. Data sharing is not applicable to this article.

## Supplementary Materials

The following are available online at https://www.mdpi.com/1999-4915/13/1/53/s1, Table S1: Membrane proteins identified by LC-MS/MS.
