# Supplementary material for: Protein Disulfide Isomerase A4 Is Involved in Genome Uncoating during Human Astrovirus Cell Entry
Source: Viruses. 2020 Dec 31;13(1):53. doi: 10.3390/v13010053 (PMC7824429; doi:10.3390/v13010053)
Supplement: Supplementary file 1 [file viruses-13-00053-s001.pdf]

viruses

MDPI

Supplementary Materials: Protein disulfide isomerase A4 is involved in genome uncoating during human astrovirus cell entry

Table 1. Membrane protein identified by LC-MS/MS.

Protein	Molecular Weight	Protein ID (Uniprot)	Caco-2	MDCK II	MA104	Vero	
Heat shock cognate 71 kDa protein (Fragment)	20 kDa	Q96IS6	X				DOI: 10.1128/JVI.76.8.4096-4102.2002
Heat shock 70 kDa protein 1A*	70 kDa	P0DMV8	X	X	X		DOI: 10.1080/22221751.2018.1557988<br>DOI: 10.1016/j.cell.2015.10.046<br>DOI: 10.1002/jmv.20786
Protein disulfide-isomerase A4*	73 kDa	P13667	X	X	X	X	
Protein disulfide-isomerase PDIA3*	57 kDa		X		X		DOI: 10.1016/j.redox.2019.101129
Junction plakoglobin	82 kDa	P14923	X	X			
Ezrin	69 kDa	P15311	X				
Sodium/potassium-transporting ATPase subunit alpha-1*	74 kDa	P05023-2	X			X	DOI: 10.1371/journal.ppat.1007963
Na(+)/H(+) exchange regulatory cofactor NHE-RF3	45 kDa	Q5T2W1-2	X				
Radixin OS=Homo sapiens	71 kDa	P35241-5	X				
Cytoskeleton-associated protein 4	66 kDa		X	X			
Isoform 2 of Syntaxin-binding protein 2	66 kDa	Q15833-2	X				
Isoform 2 of 4F2 cell-surface antigen heavy chain	58 kDa	P08195-2	X				
Lyosome membrane protein 2*	38 kDa	Q14108-2	X				DOI: 10.1038/nm.1992<br>DOI: 10.1073/pnas.96.5.2129
Plastin-2	70 kDa		X				
Isoform 2 of Basigin	29 kDa	P35613-2	X				
Heat shock protein HSP 90-beta*	83 kDa	P08238	X	X			DOI: 10.1002/jmv.20786<br>DOI: 10.1371/journal.pone.0077133
Cadherin-1	97 kDa	P12830	X				
Transferrin receptor protein 1*	85 kDa	P02786	X				DOI: 10.1073/pnas.1301764110<br>DOI: 10.1007/s00705-015-2652-3<br>DOI: 10.1128/JVI.01278-19
Isoform 2 of Myoferlin	230 kDa	Q9NZM1-2	X				
Isoform 10 of Fibronectin	240 kDa	P02751-10	X				
Neutral amino acid transporter B*	57 kDa	Q15758	X				DOI: 10.1128/JVI.73.5.4470-4474.1999

Isoform 1 of Scavenger receptor class B member 1*	57 kDa	Q8WTV0-2	X		DOI: 10.1016/j.jhep.2017.03.020
Isoform 2 of Epsin-1	69 kDa	Q9Y6I3-1	X		
Vitronectin	54 kDa	P04004	X		
Basal cell adhesion molecule	67 kDa	P50895	X		
Protein kinase C iota type	68 kDa	P41743	X		
Plasma membrane calcium-transporting ATPase 1	130 kDa	P20020-2	X		
Isoform 2 of Afadin	206 kDa	P55196-1	X		
Plexin-B2	205 kDa	PLXB2			
Poliovirus receptor*	44 kDa	F1PVS7		X	DOI: 10.1073/pnas.88.10.4104

The proteins identified for each cell line summarizes the data obtaind in three (Caco-2), two (MDCK-II), and one (MA1014, Vero) assay replicates; * Proteins previously described as virus receptors or factors important for virus entry.
